# Supplementary material for: Urgency urinary incontinence, loss of independence, and increased mortality in older adults: A cohort study
Source: PLoS One. 2021 Jan 20;16(1):e0245724. doi: 10.1371/journal.pone.0245724 (PMC7817052; doi:10.1371/journal.pone.0245724)

**S2 Figure** Results of cumulative incidence for the competing analyses. A, with or without UUI for LOI in the secondary analysis; B, with or without UUI for death in the secondary analysis; C, with mild-to-moderate UUI, severe UUI, and without UUI for LOI in the tertiary analysis; D, with mild-to-moderate UUI, severe UUI, and without UUI for death in the tertiary analysis.


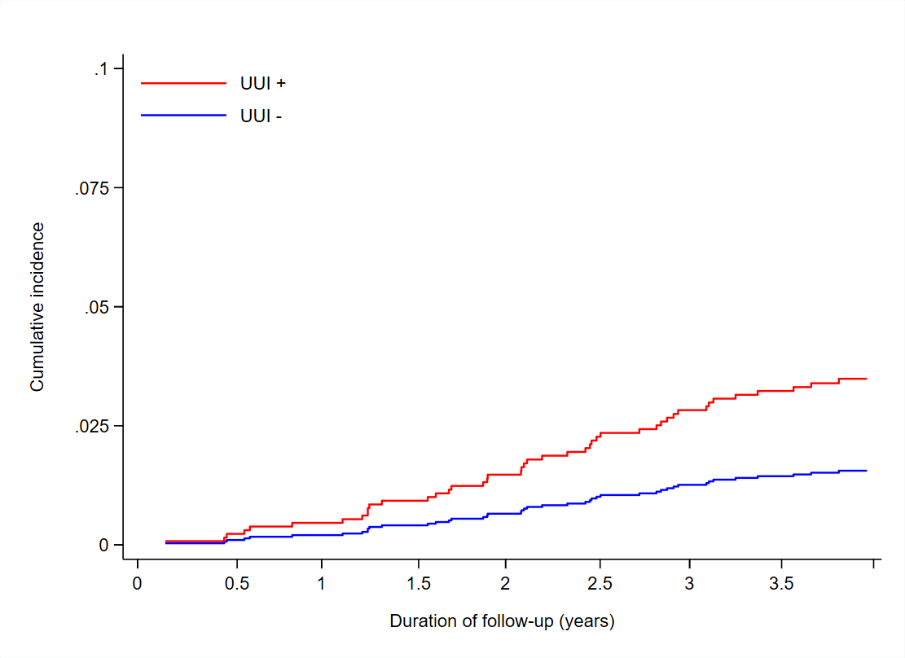

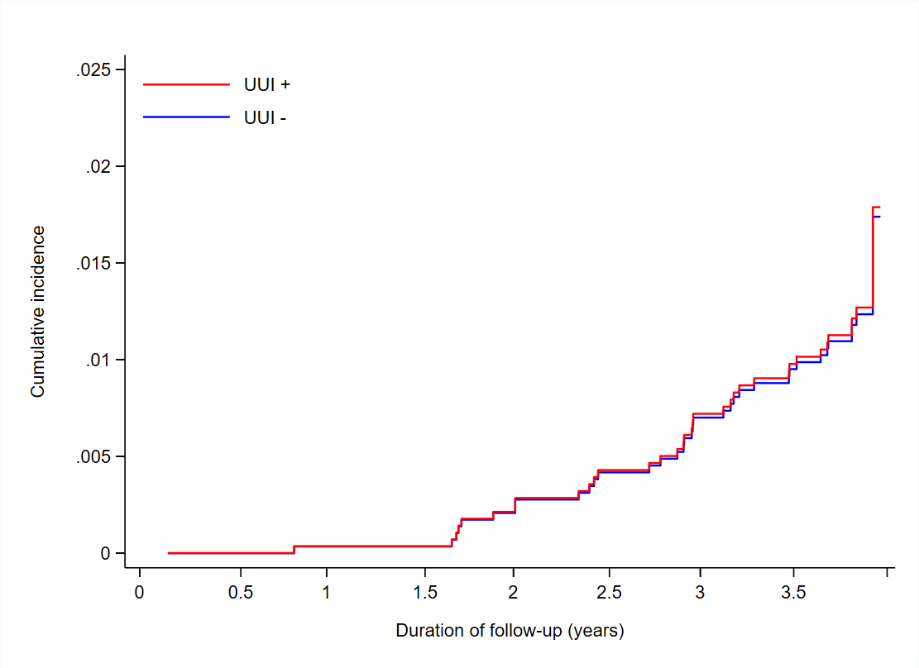
A. B.


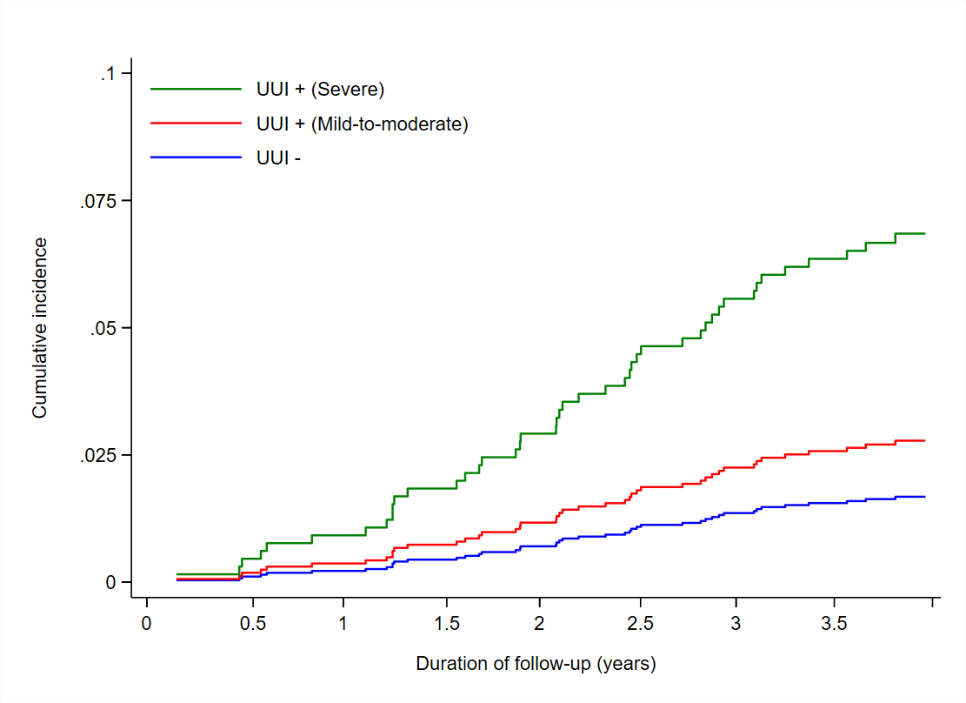
C. D.


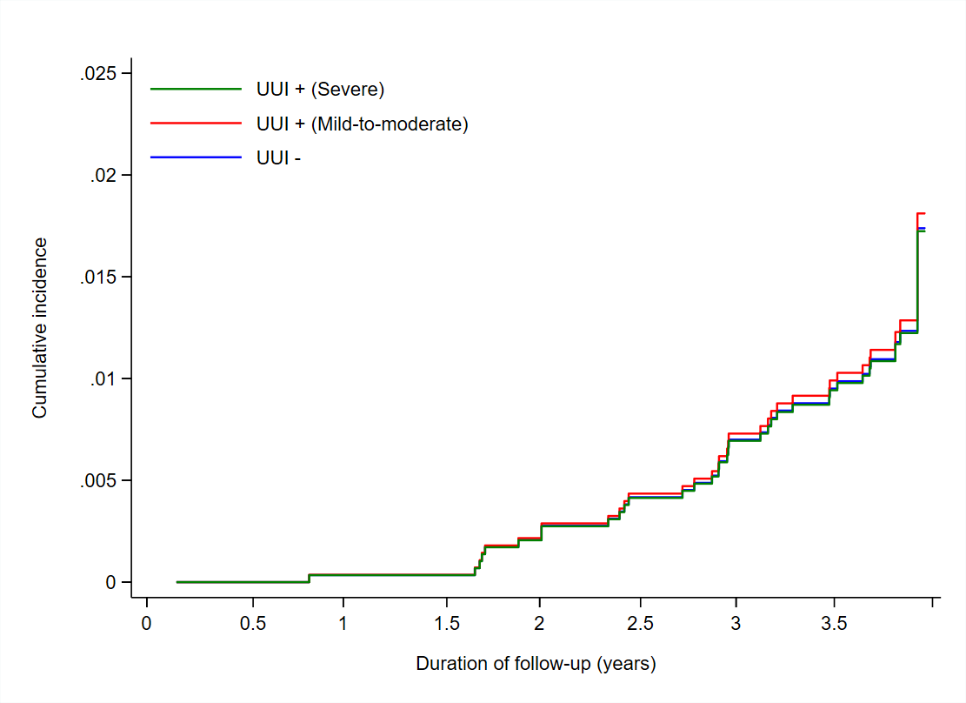

Supplement: S2 Fig — A, with or without UUI for LOI in the secondary analysis; B, with or without UUI for death in the secondary analysis; C, with mild-to-moderate UUI, severe UUI, and without UUI for LOI in the tertiary analysis; D, with mild-to-moderate UUI, severe UUI, and without UUI for death in the tertiary analysis. (DOCX) [file pone.0245724.s002.docx]
