# Supplementary material for: Urgency urinary incontinence, loss of independence, and increased mortality in older adults: A cohort study
Source: PLoS One. 2021 Jan 20;16(1):e0245724. doi: 10.1371/journal.pone.0245724 (PMC7817052; doi:10.1371/journal.pone.0245724)

**S3 Figure** Description of log-minus-log-survival plots for the evaluation of proportional hazard assumption in the tertiary analysis. A, LOI or death; B, LOI; C, death

A B


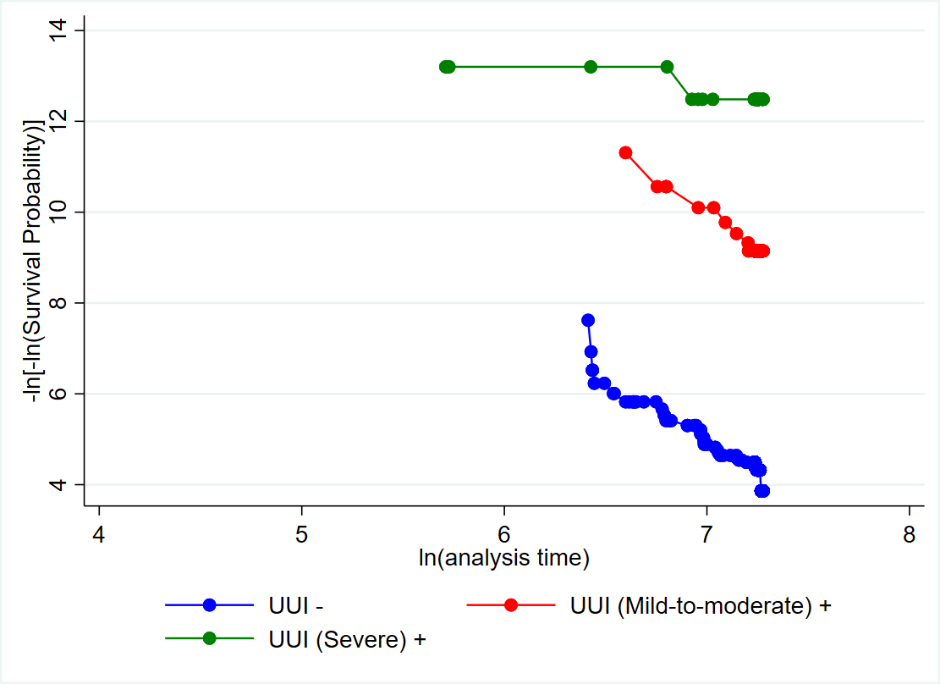

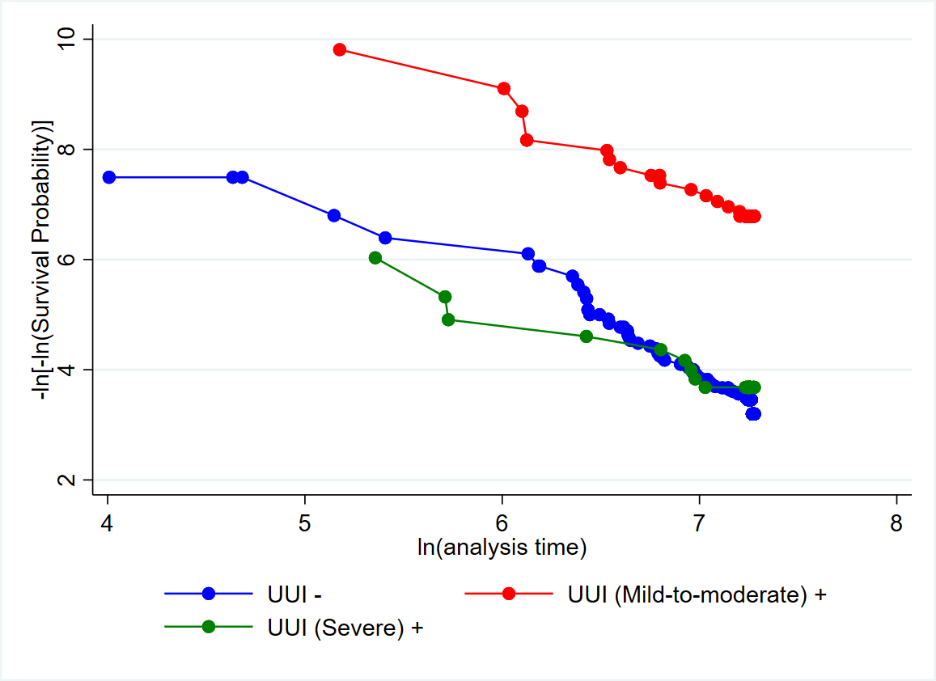


C


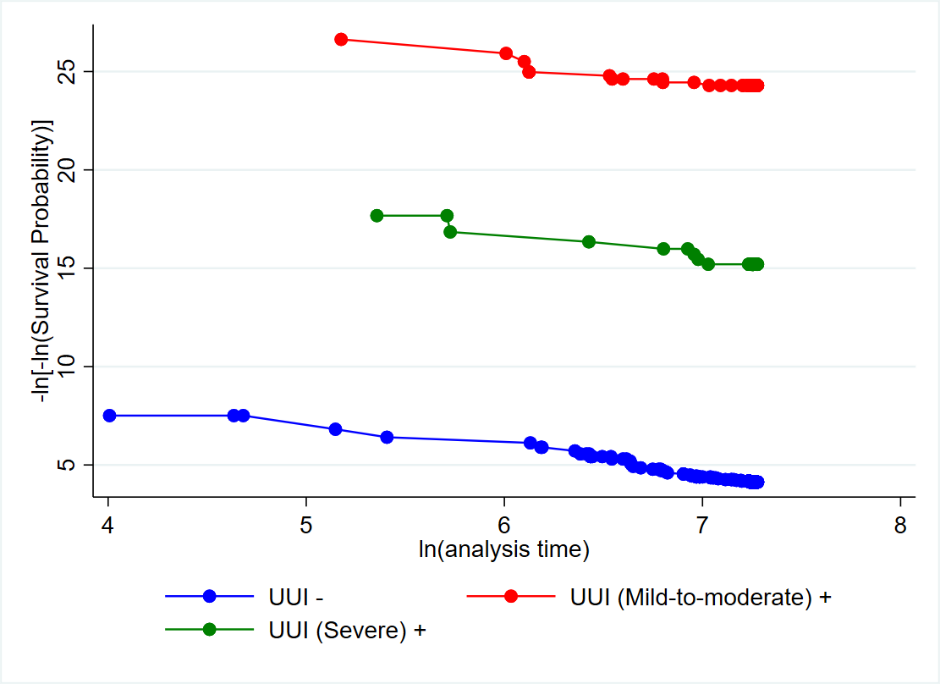

Supplement: S3 Fig — A, LOI or death; B, LOI; C, death. (DOCX) [file pone.0245724.s003.docx]
