# Supplementary material for: Urgency urinary incontinence, loss of independence, and increased mortality in older adults: A cohort study
Source: PLoS One. 2021 Jan 20;16(1):e0245724. doi: 10.1371/journal.pone.0245724 (PMC7817052; doi:10.1371/journal.pone.0245724)
Supplement: S1 Table — (DOCX) [file pone.0245724.s004.docx]

| **S1 Table** Baseline characteristics of participants classified into UUI severity | | | | | | | | | |  |  |  |
| --- | --- | --- | --- | --- | --- | --- | --- | --- | --- | --- | --- | --- |
|  |  | Total | |  | Participants | |  | Participants with | |  | Participants with | |
|  |  |  |  |  | without UUI | |  | mild-to-moderate UUI | |  | severe UUI | |
|  |  | n = 1,580 | |  | n = 1,252 | |  | n = 252 | |  | n = 76 | |
|  |  | Summary | |  | Summary | |  | Summary | |  | Summary | |
| Age (years; mean ± SD) |  | 72.8 ± 4.7 | |  | 72.5 ± 4.7 | |  | 74.2 ± 4.5 | |  | 74.3 ± 4.5 | |
| Sex (male; n, %) |  | 680 | 43.0 |  | 570 | 45.5 |  | 82 | 32.5 |  | 28 | 36.8 |
| Body mass index (kg/m^2^; mean ± SD) |  | 23.8 ± 3.0 | |  | 23.7 ± 2.9 | |  | 24.1 ± 3.1 | |  | 24.4 ± 3.3 | |
| Smoking status (current smoker; n, %) |  | 130 | 8.2 |  | 114 | 9.1 |  | 10 | 4.0 |  | 6 | 7.9 |
| Alcohol use (yes; n, %) |  | 636 | 40.3 |  | 518 | 41.4 |  | 79 | 31.3 |  | 39 | 51.3 |
| Hypertension (yes; n, %) |  | 864 | 54.7 |  | 686 | 54.8 |  | 133 | 52.8 |  | 45 | 59.2 |
| Dyslipidemia (yes; n, %) |  | 462 | 29.2 |  | 357 | 28.5 |  | 84 | 33.3 |  | 21 | 27.6 |
| Diabetes (yes; n, %) |  | 133 | 8.4 |  | 103 | 8.2 |  | 27 | 10.7 |  | 3 | 3.9 |
| History of heart disease (yes; n, %) |  | 151 | 9.6 |  | 113 | 9.0 |  | 28 | 11.1 |  | 10 | 13.2 |
| History of stroke (yes; n, %) |  | 60 | 3.8 |  | 45 | 3.6 |  | 11 | 4.4 |  | 4 | 5.3 |
| Note. UUI: urgency urinary incontinence. SD: standard deviation | | | | | | | | | |  |  |  |
