# Supplementary material for: Urgency urinary incontinence, loss of independence, and increased mortality in older adults: A cohort study
Source: PLoS One. 2021 Jan 20;16(1):e0245724. doi: 10.1371/journal.pone.0245724 (PMC7817052; doi:10.1371/journal.pone.0245724)
Supplement: S2 Table — (DOCX) [file pone.0245724.s005.docx]

| **S2 Table** Results of competing risk regression analysis | | | | | | | | | | | | | |
| --- | --- | --- | --- | --- | --- | --- | --- | --- | --- | --- | --- | --- | --- |
|  |  | LOI | | | | |  | Death | | | | | |
|  |  | SHR |  | 95% CI | | |  | SHR |  | 95% CI | | |  |
| Presence of UUI (ref. no UUI) |  |  |  |  |  |  |  |  |  |  |  |  |  |
| Model 1 |  | 1.31 |  | 0.61 | － | 2.80 |  | **2.22** |  | **1.20** | **－** | **4.10** |  |
| Model 2 |  | 1.05 |  | 0.49 | － | 2.24 |  | **2.19** |  | **1.16** | **－** | **4.13** |  |
| Model 3 |  | 1.05 |  | 0.49 | － | 2.27 |  | **2.24** |  | **1.18** | **－** | **4.27** |  |
| Model 4 |  | 1.03 |  | 0.48 | － | 2.22 |  | **2.26** |  | **1.15** | **－** | **4.44** |  |
| Presence of mild-to-moderate UUI (ref. no UUI) | | |  |  | | |  |  |  |  | | |  |
| Model 1 |  | 1.33 |  | 0.58 | － | 3.06 |  | 1.62 |  | 0.76 | － | 3.43 |  |
| Model 2 |  | 1.08 |  | 0.47 | － | 2.47 |  | 1.63 |  | 0.77 | － | 3.46 |  |
| Model 3 |  | 1.08 |  | 0.47 | － | 2.49 |  | 1.68 |  | 0.79 | － | 3.58 |  |
| Model 4 |  | 1.04 |  | 0.45 | － | 2.43 |  | 1.67 |  | 0.77 | － | 3.62 |  |
| Presence of severe UUI (ref. no UUI) | | |  |  | | |  |  |  |  | | |  |
| Model 1 |  | 1.25 |  | 0.29 | － | 5.33 |  | **4.24** |  | **1.86** | **－** | **9.69** |  |
| Model 2 |  | 0.96 |  | 0.22 | － | 4.16 |  | **3.92** |  | **1.67** | **－** | **9.24** |  |
| Model 3 |  | 0.97 |  | 0.22 | － | 4.24 |  | **3.94** |  | **1.63** | **－** | **9.55** |  |
| Model 4 |  | 0.98 |  | 0.23 | － | 4.30 |  | **4.14** |  | **1.69** | **－** | **10.1** |  |

**Note.** LOI: loss of independence. SHR: subddistribution hazard ratio. CI: confidence intervals. UUI: urgency urinary incontinence.

**Model 1** - crude model. **Model 2** - adjusted for age and gender. **Model 3** - adjusted for body mass index, smoking status and alcohol use, in addition to model 2. **Model 4** - adjusted for hypertension, dyslipidemia, diabetes mellitus, and histories of heart disease and stroke, in addition to model 3
